# Biotechnological Approaches for Genetic Improvement of Lemon (*Citrus limon* (L.) Burm. f.) against *Mal Secco* Disease

**DOI:** 10.3390/plants10051002

**Published:** 2021-05-17

**Authors:** Chiara Catalano, Mario Di Guardo, Gaetano Distefano, Marco Caruso, Elisabetta Nicolosi, Ziniu Deng, Alessandra Gentile, Stefano Giovanni La Malfa

**Affiliations:** 1 Department of Agriculture, Food and Environment (Di3A), University of Catania, via Valdisavoia 5, 95123 Catania, Italy; chiara.catalano@phd.unict.it (C.C.); mario.diguardo@unict.it (M.D.G.); distefag@unict.it (G.D.); elisabetta.nicolosi@unict.it (E.N.); stefano.lamalfa@unict.it (S.G.L.M.); 2 CREA, Research Centre for Olive, Fruit and Citrus Crops, Corso Savoia 190, 95024 Acireale, Italy; marco.caruso@crea.gov.it; 3 College of Horticulture and Landscape, Hunan Agricultural University, Changsha 410128, China; deng7009@163.com

**Keywords:** *Plenodomus tracheiphilus*, tolerance, molecular markers, phenotyping

## Abstract

Among *Citrus* species, lemon is one of the most susceptible to *mal secco* disease, a tracheomycosis caused by the mitosporic fungus *Plenodomus tracheiphilus,* which induces chlorosis followed by leaf drop and progressive desiccation of twigs and branches. Severe infection can cause the death of the plant. Since no effective control strategies are available to efficiently control the pathogen spread, host tolerance is the most desirable goal in the struggle against *mal secco* disease. To date, both traditional breeding programs and biotechnological techniques were not efficient in developing novel varieties coupling tolerance to *mal secco* with optimal fruit quality. Furthermore, the genetic basis of host resistance has not been fully deciphered yet, hampering the set-up of marker-assisted selection (MAS) schemes. This paper provides an overview of the biotechnological approaches adopted so far for the selection of *mal secco* tolerant lemon varieties and emphasizes the promising contribution of marker-trait association analysis techniques for both unraveling the genetic determinism of the resistance to *mal secco* and detecting molecular markers that can be readily used for MAS. Such an approach has already proved its efficiency in several crops and could represent a valuable tool to select novel lemon varieties coupling superior fruit quality traits and resistance to *mal secco*.

## 1. Introduction

Lemon (*Citrus limon* (L.) Burm. f.) is the third crop for economic importance among citrus species. Together with lime, their global harvested area amounts to 1.2 million hectares with a corresponding production of 20.1 million tons (FAOSTAT, 2019). The 48% of the global lemon production comes from the Mediterranean Basin and the Black Sea area. In these areas, lemon cultivation is threatened and severely limited by a devastating vascular disease named *mal secco*, which means ‘dry disease’ in Italian. Its complex symptomatology consists of leaf vein chlorosis, phylloptosis, wood discoloration, necrosis, leading to the progressive desiccation of the whole plant. The causative agent of *mal secco* disease is the mitosporic fungus *Plenodomus tracheiphilus* (Petri) Gruyter, Aveskamp, and Verkley (syn. *Phoma tracheiphila* (Petri) Kantschaveli and Gikashvili), included in the A2 list of quarantine pests of the European and Mediterranean Plant Protection Organization (EPPO). Lemon is the most susceptible species among citrus and economic losses related to *mal secco* disease are dramatic for the Mediterranean’s citrus industry [1]. In fact, *mal secco* disease has a direct impact on the production volumes, and several indirect impacts related to the very high costs related to the disease control (pruning of affected branches and replanting of dead plants). Moreover, the tolerant cultivars are characterized by poor fruit quality thus reducing the economic value of the marketable lemons. Currently, both chemical and agronomic measures are not sufficient to contain the diffusion of the pathogen raising the interest for the elucidation of the host tolerance mechanism against *mal secco*. It threatens other species and cultivars introduced in the agricultural system (e.g., the mandarin ‘Cassar’ and the sweet orange ‘New Hall’ in Tunisia) [2].

In the last decade, many authors provided valuable reviews on the lemon susceptibility to *mal secco* focusing on the pathogen and/or the host response [3,4]. Nevertheless, many aspects, especially the mechanisms of the host-pathogen interaction are not fully understood [5]. More recently, a complete review has been published describing the strategies pursued to select lemon genotypes with enhanced tolerance to *mal secco* disease [6]. Biotechnological approaches for lemon breeding need to be reviewed in-depth since they represent a cost and time-effective strategy toward the selection of tolerant citrus genotypes [7]. This is particularly relevant in light of the fast improvement in the biotechnological field (both in terms of throughputs and technology).

Traditional breeding (mass, clonal and nucellar selection, hybridization, mutagenesis) enabled the obtainment of several novel lemon varieties [3,4,5,6,8]. Clonal selection improved field tolerance to the disease. However, field-tolerant varieties are usually less productive, and their fruits have lower quality in terms of fruit size, acidity, or juice content. Mutagenesis was not successful in generating tolerant or resistant varieties. Hybridization has been extensively used in breeding programs, but it is extremely difficult to obtain *mal secco*-resistant lemon hybrids with fruit shape, flavor, and aromas comparable to those of a true lemon. Therefore, none of the cultivars generated by conventional breeding approaches combined tolerance to *mal secco* disease, high yield, fruit quality, and off-season production [4]. Biotechnological strategies such as in vitro selection, somatic hybridization, and genetic transformation can instead represent promising strategies to select genotypes showing good tolerance to the disease and overcome the limitations of traditional breeding approaches.

The development of high-throughput sequencing platforms enabled the set-up of whole-genome resequencing projects; Russo and colleagues [9] presented an RNAseq experiment on rough lemon (*C. jambhiri*, susceptible to *mal secco*) leading to the identification of two candidate genes: RPM1 interacting protein 4 and BIR2, that can be further validated through genome editing experiments.

**Figure 1:**
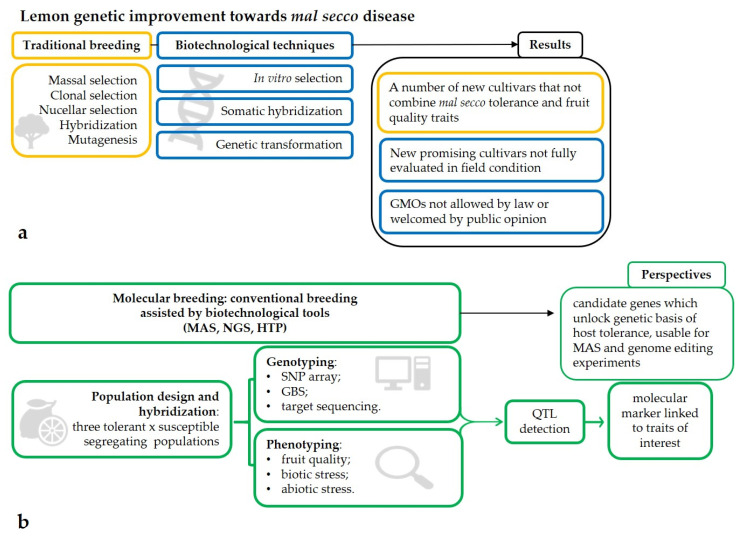
A schematic view of lemon breeding techniques used in the past and supposed to be used in the next future to obtain genotypes with enhanced resistance to *mal secco* disease and their respective results: (**a**) traditional and biotechnological techniques led to the obtainment of new selections which did not combine *mal secco* tolerance and fruit quality traits, whose tolerance has not been fully demonstrated in field conditions, or which use is hampered because Genetically Modified Organisms; (**b**) molecular breeding strategies combining hybridization and high-throughput genotyping and phenotyping in a marker-trait association analysis will hopefully lead to the identification of candidate genes linked to *mal secco* tolerance.

In the present review, biotechnological approaches for lemon breeding against *mal secco* disease will be deeply covered, with the final aim of showing the potential of molecular markers to develop novel breeding strategies leading to the detection of resistant varieties that can meet the consumers’ standards (Figure 1).

## 2. Biotechnological Approaches

### 2.1. In Vitro Selection

In vitro selection has been the first biotechnological approach ever pursued to improve the efficiency of traditional citrus breeding programs for the selection of *mal secco* tolerant genotypes. This approach is aimed at the identification of somaclonal variation naturally occurring in protoplast and calli were grown in selective tissue culture. Plant cells are thus grown in vitro in a tissue culture media in which phytotoxic compounds produced by the fungus are added as selective agents. Such technique enables the screening of hundreds of protoplasts or embryogenic calli (from which whole plants can be regenerated) simultaneously and guarantees a high control of the environmental variability since plant tissues are maintained in growth chambers [10].

The fungal culture of *P. tracheiphilus* produces a complex of glycoproteins named *malseccin* [11]. In lemon, two fractions (with molecular weight equal to 93 kDa [12] and 60 kDa [13] respectively) showed the capacity to induce symptoms in leaves comparable to those of *mal secco* suggesting their involvement in pathogenesis. Another fraction with a lower molecular weight (350–700 Da) was also isolated and later named *mellein*, but its phytotoxic activity on lemon leaves was not demonstrated due to its low concentration in *P. tracheiphilus* culture filtrate. *Mellein* is thus likely involved in symptoms development in synergy with other phytotoxic metabolites produced by the pathogen [14,15].

Since the identification of such phytotoxic compounds, a number of researches were conducted to (1) investigate their translocation and effect in infected tissues of lemon [16,17,18,19,20], (2) understand the potential correlation between toxins and the different degree of virulence among *P. tracheiphilus* strains [21,22], and (3) verify the reliability and efficiency of these substances for the screening of citrus genotypes by treating cell cultures, seeds, seedlings, young shoots, cuttings and leaf discs with crude culture filtrate or with the partially purified toxin (PPT) [23,24,25,26,27,28,29].

Nadel et al. [30] reported the first experiment of in vitro selection of cell lines of ‘Villafranca’ lemon showing tolerance to *mal secco* toxins. Calli underwent a rigorous selection protocol and a selected line (Variant 1.117) showed trait stability after three subcultures on non-selective media and the shift from a non-differentiated state (callus) to a differentiated state (somatic embryos) and vice versa.

Later, Gentile et al. [31,32,33,34,35,36] realized a comprehensive study for in vitro selection of new lemon cultivars with improved tolerance to *mal secco* through a strategy consisting of (1) identification of the appropriate selecting agent for in vitro selection [31], (2) regeneration of somaclonal variants under the appropriate selecting media [32], and (3) analysis of the metabolites produced by these variants to verify the mechanism of tolerance [33,34,35,36].

As mentioned above, the first step for the optimization of an in vitro selection experiment regards the choice of the appropriate selecting agent [10]. In a pilot study, the response of nucellar calli and protoplasts from two species with different tolerance to *mal secco* disease (the tolerant sweet orange, *C. sinensis*, and the susceptible lemon) was tested under the effect of both the culture filtrate (CF) and the partially purified toxin (PPT) produced in culture by the pathogen [31]. Results showed that lemon calli and protoplasts underwent a much higher growth compared to orange in a medium containing 50% of CF. Such unexpected results can be because CF contains kinetin and gibberellic acid (GA3) in very low concentration, and a relatively high level of indole-3-acetic acid (IAA). As a consequence of different sensitivity of the two genotypes to the presence of these hormones, sweet orange and lemon protoplasts responded in reverse order to the exposure to CF than expected with reference to *mal secco* tolerance. On the other hand, as expected, under the effect of PPT, thus without the effect of the hormones accumulated in the CF, lemon callus and protoplasts were much more affected than those of sweet orange.

Since PPT was proved to be more efficient as a selective agent for in vitro selection experiments, calli from the susceptible lemon ‘Femminello Continella’ were treated with sublethal doses of PPT leading to the detection of a toxin-tolerant cell line named ‘Femminello-S’ [32]. To further validate this finding, protoplasts from 50 ‘Femminello-S’ regenerated lines were treated with PPT and showed a relevant proportion of tolerant genotypes, compared to other susceptible lemon varieties.

**Figure 2:**
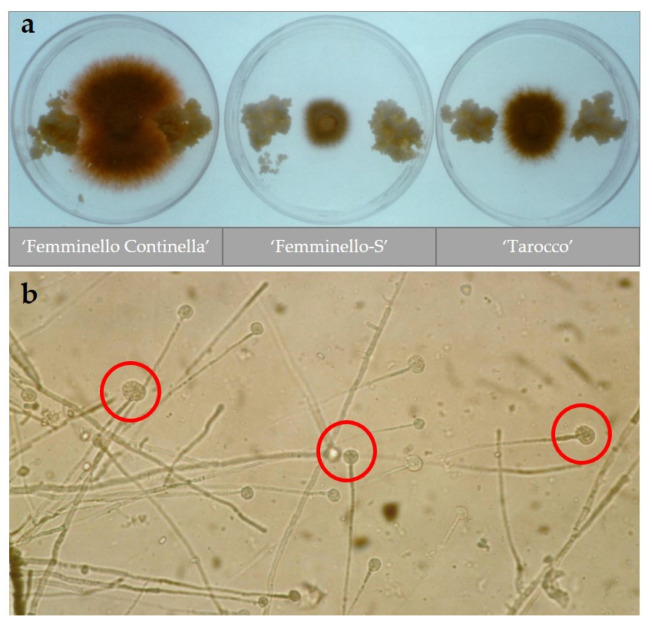
In vitro activity of ‘Femminello-S’ lemon callus. (**a**) *Plenodomus tracheiphilus* growth on MT medium in the presence of 30-day-old citrus calli (from left: ‘Femminello Continella’ lemon, ‘Femminello-S’ lemon, and ’Tarocco’ sweet orange). Calli were cultured for 30 days and then inoculated and co-cultured with the fungus for 10 days. The tolerant genotypes ‘Tarocco’ and ‘Femminello-S’ showed inhibition of the fungal growth, in contrast to what was observed for ’Femminello Continella’ lemon. (**b**) *P. tracheiphilus* hyphae tips lysing after 5 minutes from the application of 10 µg of proteins extracted from the culture medium of ‘Femminello-S’ lemon somaclone (circled in red). Adapted from Gentile et al. [34].

Dual-culture of calli from different citrus genotypes grown with *P. tracheiphilus* mycelium (Figure 2a) and the analysis of extracellular proteins produced by the calli and accumulated in the conditioned media were also performed to understand the different behavior of the ‘Femminello-S’ line compared to the control [34]. It was shown that ‘Femminello-S’ has a ten-fold increase of two hydrolase proteins related to pathogenesis (PR-proteins), chitinases, and (1–3)-β-glucanase, compared to ‘Femminello Continella’. Moreover, it was demonstrated that 10 µg of chitinases are sufficient to cause the lysis of the fungal hyphal tips (Figure 2b), suggesting a positive correlation between ‘Femminello-S’ tolerance to *P. tracheiphilus* toxin and the release of chitinases and glucanase in its culture media.

Following this research line, an immunoenzymatic assay was conducted by treating, with specific chitinase antibodies (from tobacco and lemon), extracellular proteins from cellular suspension, and leaves of a number of genotypes with known behavior towards *mal secco* disease [34,35]. The presence of 34 and 45 kDa proteins in leaf cellular suspension suggested the involvement of several chitinases synthesized by different genes. Moreover, a higher reaction to a polypeptide of 34–40 kDa was registered in tolerant genotypes compared to the susceptible ones. Finally, when production and accumulation of PR-proteins were investigated in tolerant (‘Murcott’ tangor) and susceptible (‘Messina’ lemon) citrus genotypes grown under the effect of PPT, the tolerant genotype showed an earlier production of such compounds compared to susceptible ones.

‘Femminello-S’ lines were also tested in vivo for tolerance to *mal secco* infection and showed mild symptoms, comparable to what is observed for the resistant lemon ‘Monachello’ [37,38]. Under field conditions, in areas where *mal secco* is endemic, these cultivars showed a partial tolerance, but an in-field characterization has never been completed and ‘Femminello-S’ has never been registered as a new lemon variety. Recently, Russo and co-workers [39] reported mild to severe *mal secco* symptoms for ‘Femminello-S’ plants included in their in-field characterization in an area of high pathogen pressure.

Since it was demonstrated that in vitro selection of callus and protoplasts with *P. tracheiphilus* toxin was an effective tool to detect tolerant genotypes, a new experiment on protoplast was performed [40]. The advantage of using protoclones (thus plants derived from a single cell) compared to somaclones (derived from callus) is that no escape events can occur [32]. This work enabled the selection of two protoclones whose tolerance was comparable to that of the tolerant ‘Monachello’ [40].

The last in vitro selection experiment reported was performed by Bas and co-workers [41]. They selected a stable resistant callus mass ‘20 b’ from the susceptible lemon ‘Kütdiken’ that strongly inhibited the fungal growth of *P. tracheiphilus*, as well as other microorganisms (*Phytophthora citrophthora*, *Geotrichum candidum*, *Diplodia natalensis*) in co-culture experiments.

The in vitro selection proved its efficacy in selecting tolerant genotypes and for the investigation of the plant response to the toxin produced by the pathogen. Unfortunately, a complete characterization of the selected genotypes under field conditions has never been performed, except for the recent work by Russo et al. [39], and more research on the role of phytotoxic metabolites in pathogenesis and on plant physiology are still needed. Researches concerning PR-proteins did not fully clarify their role in tolerance mechanisms towards *mal secco* disease (e.g., characterization of the constitutive chitinases of lemon and its amino-acid sequence), even though they opened the way to genetic transformation experiments. In fact, chitinase from *Trichoderma harzianum* was used in an *Agrobacterium*-mediated transformation experiment to obtain *mal secco* tolerant lemons [42,43].

### 2.2. Somatic Hybridization

Somatic hybridization consists of the fusion of protoplasts originating from embryogenic callus, cell culture suspension, or leaves. This hybridization results in a new genotype originating from the combinations of the genomes of the two protoplasts. This biotechnological strategy is useful to develop novel selections overcoming sexual incompatibility, nucellar polyembryony, long juvenility, and pollen and ovule sterility [44]. Other applications of somatic hybridization are the production of tetraploid genotypes that can be employed as parental lines in interploid crosses for obtaining seedless triploids, or the direct production of triploids by fusion of haploid and diploid protoplasts [45].

As reviewed by Grosser and Gmitter [46], the somatic hybridization approach for citrus breeding showed some limitations, such as (1) at least one of the two parents should be embryogenic in vitro (able to produce somatic embryos), limiting the number of genotypes available for the experiments, (2) the hybrids obtained could be infertile, (3) each pair of parents result in only one progeny, because segregation and recombination depend from sexual propagation, and (4) subsequent sexual hybridizations are needed to eliminate negative traits or to generate seedless triploids genotypes. Tusa and collaborators [47] have been the first to apply somatic hybridization to obtain lemon-like genotypes with increased tolerance towards *mal secco* disease. Interspecific somatic hybrids of ‘Valencia’ sweet orange and ‘Femminello’ lemon were generated via somatic embryogenesis following protoplast fusion through the polyethylene glycol method to combine the cold hardiness and *mal secco* tolerance of ‘Valencia’ with the optimal fruit quality and productivity of ‘Femminello’. In later experiments, new allotetraploid somatic hybrids were generated combining ‘Hamlin’ sweet orange or ‘Milam’ lemon (a selection of rough lemon, *C. jambhiri*) with ‘Femminello’ lemon, then, the allotetraploid somatic hybrids were crossed again with the diploid ‘Femminello’ lemon to obtain triploids, seedless, plants [48,49]. In a work by Grosser et al. [45], several asymmetrical somatic hybrids were generated to understand the factors involved in the regeneration of hybrids (cybrids) containing the nucleus of one parent and some organelles of the latter.

Hybrids obtained by symmetrical (‘Valencia’ sweet orange + ‘Femminello’ lemon) and asymmetrical (triploid and tetraploid cybrids of ‘Femminello’ lemon) protoplasts fusion were tested for *mal secco* infection by stem and leaf inoculation [50]. In particular, ‘Valencia+Femminello’ hybrids showed a slower development of symptoms, a lower rate of propagules in the xylem, and a lower percentage of dead plants compared to the susceptible control. This different behavior is probably due to the additive effect of symmetrical protoplast fusion on traits whose expression depends on complex gene patterns. Once again, these promising results, obtained in controlled conditions, have not been validated in open field experiments.

Somatic hybridization has been also combined with in vitro selection to detect tolerant hybrids generated from the fusion of the protoplasts of two parents (one showing resistance to *mal secco* and the other characterized by high-quality fruits). Such an approach has been carried out on ’Murcott’ tangor exposed to *P. tracheiphilus* toxin and ‘Messina’ lemon [40]. In a later study [51], RAPD (Random Amplified Polymorphic DNA) markers were also developed to early identify lemon mutants and improve the fusion efficiency. Finally, a complete proteomic and metabolomic study on orange and lemon diploid cybrids, aimed to understand nucleus-cytoplasm interaction, revealed the over-expression of a peroxidase 3-like protein, associated with the biosynthesis of syringyl lignin, which in turn is involved in disease resistance mechanism [44]. This represented a new step towards the understanding of the genetic basis of *mal secco* disease tolerance.

### 2.3. Genetic Transformation

One more attempt carried out to avoid the limitations of conventional breeding was represented by genetic transformation, which enabled the obtainment of many varieties and rootstocks with enhanced agronomic traits [52,53,54,55,56]. Through an *Agrobacterium*-mediated transformation approach, the chitinase gene *chit42* from *T. harzianum* has been transferred in ‘Femminello Siracusano’ lemon [42]. From these experiments, two transgenic lemon clones, E23 and E24, were generated with one and two copies of *chit42*, respectively.

E23 and E24 genotypes were tested for *mal secco* and *Botrytis cinerea* infection, which causes mold in fruits or a premature flower fall. Taking advance from the in vitro activity of chitinase transferred in lemon transgenic clones, assays for *mal secco* tolerance were performed in controlled conditions evaluating leaf protein effects on conidia germination and hyphae growth, thus avoiding any limitation of *in planta* artificial inoculation [57]. On the other hand, disease tolerance assays for *B. cinerea* were performed *in planta*. In both cases, lemon transgenic clones showed an enhanced tolerance toward both pathogens compared to the control. Moreover, the transgenic clones showed a higher production of the endochitinase enzyme. When also glucanase and esochitinase were tested, the E23 clone especially revealed a significantly higher expression than the control, suggesting the involvement of other genes in the plant tolerant response to the pathogen. In addition, E23 and E24 lemons did not show any morphological differences compared to the wild type, differently to what was observed in apples where transgenic plants for the same gene exhibited reduced vigor [58].

Expression patterns of defense-related genes were also evaluated in leaves of transgenic lemon clones. Under the elicitation of *B. cinerea* infection, *chit42*, endogenous chitinase, and glucanase, involved in the systemic acquired resistance (SAR), and phenylalanine ammonia-lyase (PAL) together to allene oxidase synthase (AOS), involved in induced systemic resistance (ISR), were considered [59]. In transgenic clones, a decrease of SAR-related genes has been detected, while ISR-related gene expression was significantly higher than the control. These results suggested *chit42* overexpression could improve the ISR mechanism. In Distefano et al. [60], where expression analysis was extended to phospholipid hydroperoxide glutathione peroxidase (GPX1) and fatty acid hydroperoxidase lyase (HPL), ISR plant defense increase was confirmed for transgenic lemon clones.

The same transformation experiment was replicated on Troyer citrange, where transgenic plants showed a higher chitinase and glucanase activity, as presumed, and reduced susceptibility towards crude filtrates of *P. tracheiphilus* [61].

**Figure 3:**
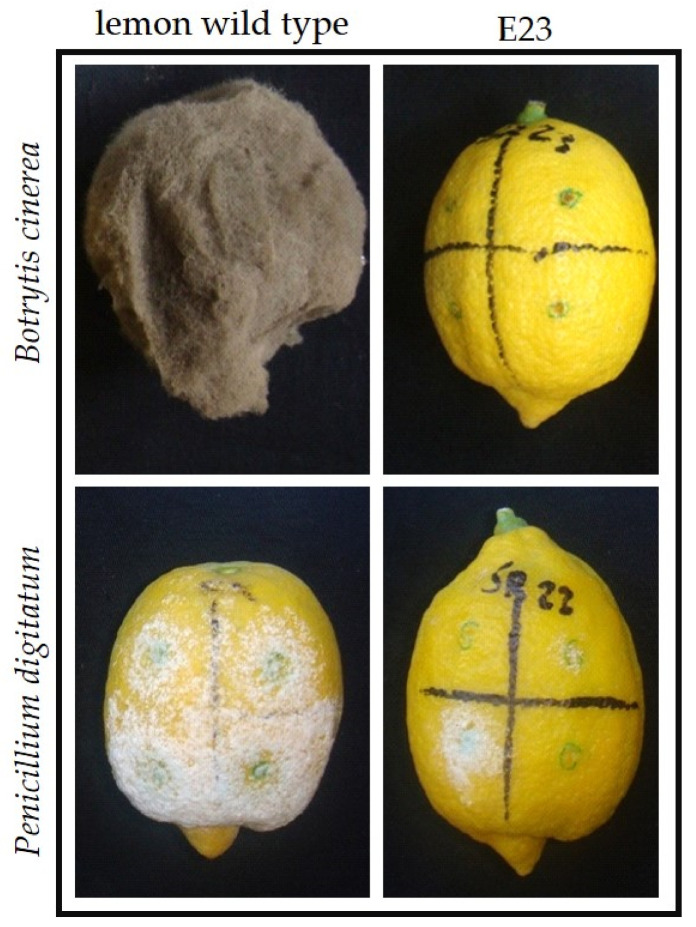
Disease symptoms in E23 transgenic lemon fruit compared to the wild type after inoculation with *Botrytis cinerea* (above) and *Penicillium digitatum* (below), after 14 and 6 days, respectively. Adapted from Oliveri et al. [62].

Further studies [62] were performed to verify the improved tolerance of transgenic lemon fruits to other fungal pathogens, such as *Colletotrichum gloeosporioides*, *Penicillium digitatum*, and *P. italicum* (Figure 3), highlighting the contribution of the genetic transformation approach to control the most important pathogens affecting lemon fruits during post-harvest storage.

The E23 transgenic clone obtained by Gentile et al. [42] has been used for a metabolomic study comparing GM and non-GM fruits, showing different tolerance to post-harvest pathogens, using a nuclear magnetic resonance-based (NMR) approach [63]. The results revealed the substantial equivalence of nutrients and toxicants between GM and non-GM lemons.

In contrast with genetic transformation promising results, GM lemon clones are not widely spread.

## 3. New Biotechnological Approaches: The Era of NGS (Next Generation Sequencing) and MAS (Marker-Assisted Selection)

To date, most of the works were focused on selecting lemon varieties with improved tolerance to *mal secco* disease through in vitro approaches, while only a few studies were focused on deciphering the genetic basis of resistance/tolerance.

Reforgiato Recupero et al. [64] performed an analysis of the genetic basis of the resistance to *mal secco* combining field phenotyping and PR proteins production (chitinase) evaluation on several progenies of *Citrus* species and *Poncirus trifoliata,* detecting three alternative genes (A, B, and C) able to determine the dominant tolerant phenotype and a fourth gene, gene D, that in the condition of dominance was able to determine susceptibility by nullifying allele B action.

A transcriptomic approach using the suppression subtractive hybridization method (SSH) was employed on ‘Femminello-S’ lemon grown in a media with *P. tracheiphilus* toxin to detect differentially expressed genes (DEG) related to plant stress response, but results led only to DEGs involved in other biochemical pathways such as plant growth and development [65].

Similarly, the SSH method was also applied by Koutsioumari et al. [66] to identify genes involved in resistance to biotic and cold stress, comparing ‘Adamopoulou’ (tolerant) and ‘Lisbon’ (susceptible) lemon varieties. This approach allowed the detection of several candidate genes such as allantoinase, cytochrome P450, 4-coumarate-CoA ligase, polyphenol oxidases, betaine aldehyde dehydrogenase, acetyl-CoA carboxyltransferases, and ultraviolet-B-repressible protein, all involved in metabolic responses to biotic and abiotic stresses.

More recently, RNA-seq analysis of the susceptible rough lemon (*C. jambhiri*) inoculated with *P. tracheiphilus* led to the identification of promising candidate genes useful for lemon breeding: RPM1 interacting protein 4, a positive regulator of plant defense, and BIR2, a negative regulator of the basal level of immunity, that was respectively down- and up-regulated in the inoculated sample, explaining the susceptibility of rough lemon towards *mal secco* infections [9].

In the last decades, the genotyping platforms experienced a tremendous leap forward in terms of throughputness, cost-effectiveness, and reliability. To this extent novel studies based on genome or transcriptome whole sequencing (e.g., whole-genome sequencing, WGS) have become a promising option to underpin the genetic determinism of a trait of interest.

The availability of high-throughput genotyping platforms is a fundamental step toward the set-up of marker-trait association analysis and the identification of molecular markers linked to traits of agronomical interest that can be used for marker-assisted selection (MAS). Among marker-trait association analysis, genome-wide association study (GWAS) approaches proven their efficacy in Citrus in the identification of genomic regions associated with fruit quality traits (e.g., weight, peeling attitude, color, texture) [67,68].

**Figure 4:**
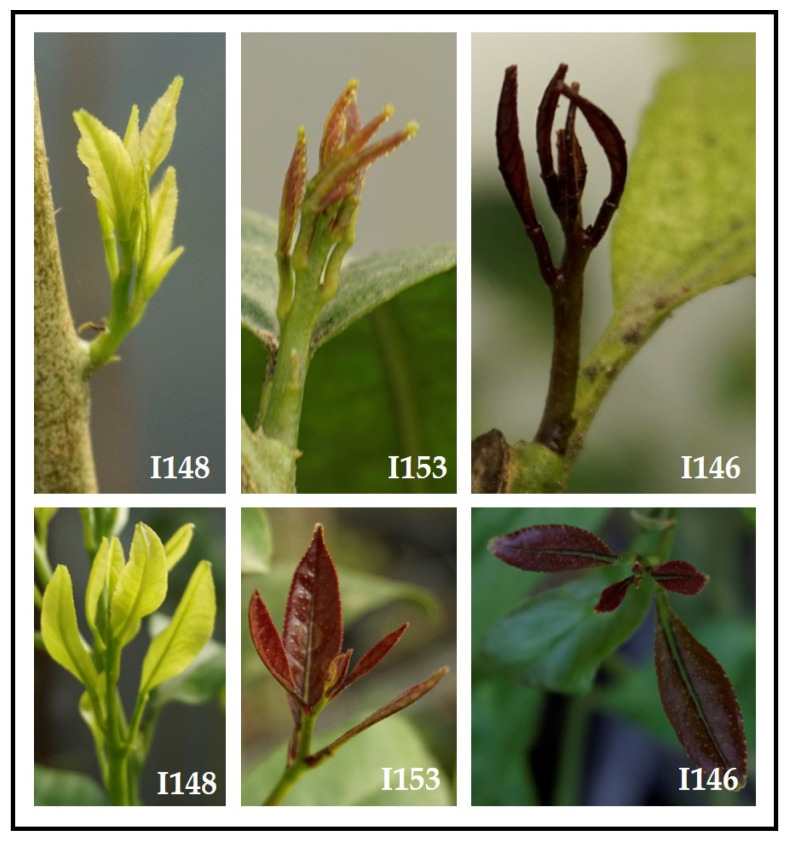
Example of character segregation in the population ‘Interdonato’ lemon × ‘Femminello Siracusano 2kr’ lemon: anthocyanin pigmentation in young shoots of three different hybrids (from left: I148, I153, and I146) in two different development stages.

**Figure 5:**
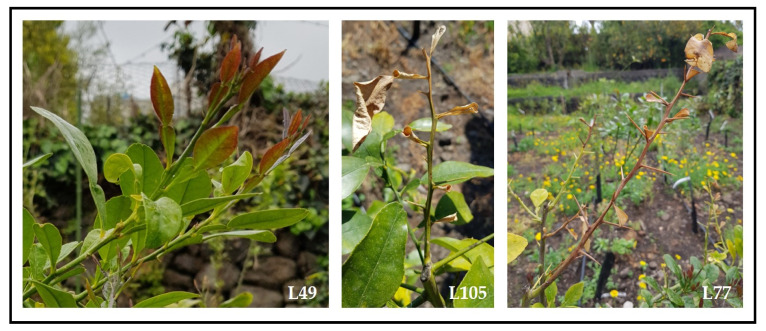
*Mal secco* symptoms on different *C. latipes* × ‘Femminello Siracusano 2kr’ lemon hybrids cultivated in an area where pathogen pressure is naturally high. From left to right: hybrid L49 does not show any symptom, hybrid L105 is at the initial phase of stem desiccation, while hybrid L77 shows complete twig dieback.

To explore the potentiality of marker-trait association and WGS approaches, novel studies are currently carried out within the framework of the projects entitled ‘Development of Resistance Inductor against Citrus Vascular Pathogens’ (S.I.R.P.A., http://www.progettosirpa.it/home) and ‘Fruit Crops Resilience to Climate Change in the Mediterranean Basin’ (FREECLIMB, https://primafreeclimb.com/). To this extent three intra- or intergeneric segregating populations were developed by crossing ’Femminello Siracusano 2kr’ (male parent: optimal fruit quality and high susceptibility to *mal secco*) with three tolerant parents namely ‘Interdonato’ lemon (130 seedlings, Figure 4), *C. latipes* (150 seedlings, Figure 5), and *C. clementina* (130 seedlings). The three full-sib populations were genotyped with a target sequencing approach (single primer enriched technology, SPET) leading to the interrogation of 50K SNPs that were polymorphic in at least one of the three populations. The SNP design has been carried out through the resequencing of the four parental lines, then the genome was divided into uniform windows (bin) of 100 Kb and 7 to 10 SNPs were selected within each bin. The probes designed on the selected SNPs were used to genotype the progenies. The SNP discovery is based on the sequencing information of the parental lines, and the availability of a high-quality reference genome is a prerequisite for the SPET analysis. Since no reference genome is publicly available for lemon (www.citrusgenomedb.org, www.phytozome.jgi.doe.gov, http://citrus.hzau.edu.cn/orange/), the *de novo* sequencing of the ‘Femminello Siracusano’ was also performed in parallel with the SPET analysis (Di Guardo, personal communication).

Marker-trait association analyses are based on the simultaneous analysis of genotypic and phenotypic data; in light of this, the three full-sib populations will be artificially inoculated, and the development of the symptoms will be monitored (see next paragraph). QTL analysis will be performed both on each full-sib family alone (single-family QTL approach) and using a combined approach called pedigree-based analysis (PBA, [69]) thanks to the fact that the three crosses have ‘Femminello Siracusano 2kr’ lemon as a common parent.

Both QTL analysis approaches will lead to the detection of genomic regions significantly associated with tolerance to *mal secco*. Molecular markers in strong linkage disequilibrium (LD) with the traits will be further validated on citrus germplasm showing a different genetic background to test marker transferability. Furthermore, the genomic regions underlying the QTL will be in silico annotated to detect candidate genes in close LD with significant SNP(s). The detection of robust marker(s) linked to *mal secco* tolerance can then be used for MAS breeding approaches to detect novel selection combining optimal fruit quality traits and improved tolerance to *mal secco*.

In *Citrus*, Genotyping By Sequencing (GBS, [70]) approach has been already performed for the detection of QTLs associated with Huanglongbing (HLB) tolerance, one of the most devastating diseases for citrus caused by *Candidatus* Liberibacter, on an intergeneric hybrid population of sweet orange (susceptible) and trifoliate orange (*P. trifoliata*, highly tolerant) [71]. This work represents a starting point for the identification of further genomic regions and genes involved in resistance against HLB. Such candidate genes can then be tested through genetic engineering and genome editing approaches. The same promising results are hopefully expected for the lemon—*mal secco* system.

## 4. Phenotyping as a Key Tool for Genotyping

It is worth noting that many authors defined phenotyping as the bottleneck for functional genomic studies and crop improvement [72,73,74,75,76,77,78]. If phenotyping is merely the assessment of morphological and physiological plant traits, phenomics, instead, is defined as the acquisition of high dimensional phenotypic data on an organism-wide scale [79], and the reliability of the marker-trait association studies is largely influenced by the quality of the phenotypic data. Large-scale genetic and breeding experiments take advantage of computer-image analysis integrated with biological data [80] such as different image techniques (visible light, fluorescence, near-infrared, hyperspectral, 3D, laser, magnetic resonance, positron emission detectors for short-lived isotopes (PET), X-ray computed tomography and X-ray digital radiography) [81,82]. Much interest is also shown toward machine learning approaches to detect patterns and hidden correlation from large amounts of data [83].

Regarding disease resistance phenotyping, the most significant improvement consisted in the passage from the traditional visual inspection and disease severity assessment through a discrete scale, to quantitative high-throughput automated image-based methods for studying how host physiology is affected by pathogens and pests [84]. The application of high-throughput phenotyping (HTP) to pathology is a recent advancement, probably because the genetics of resistance to major pathogens are relatively straightforward [72].

The phenotyping of tracheomycosis is particularly challenging since the pathogen progressively colonizes the host vascular system without developing externally visible symptoms. As for *mal secco*, another issue concerning tolerance phenotyping is that even the most tolerant species show symptoms when artificially inoculated since inoculum easily reaches xylem vessels and spreads throughout the plant.

Up to now, *mal secco* disease phenotyping has always been performed through empirical scales evaluating symptoms severity (such as vein chlorosis and stem desiccation, despite they are not specific symptoms), both in the field [39] or in controlled conditions [38], after artificial inoculation or taking advantage of the in-field natural pressure of the pathogen [50,85,86,87,88].

A digital method for *mal secco* symptoms assessment would be desirable to quantify more accurately *mal secco*—associated vein chlorosis and consequently support future breeding experiments. However, at the moment the detection of the fungus based on visual assessment is rather difficult due to the aspecificity of the disease symptoms. Image analysis and other HTP methods would be more accurate and precise, suitable for phenotypic and genotypic data crossing, even though more time-consuming [89]. Digital imaging on citrus species has been already performed by Bock et al. [90] and Pourreza et al. [91]. In the first work, citrus canker disease symptoms (caused by *Xanthomonas axonopodis* pv. *citri*) were assessed on grapefruit leaves comparing digital imaging (through Assess software) to visual measurement and demonstrating how image analysis is inexpensive and more reliable for monitoring epidemics and plant response. In the latter work, an affordable vision-based sensing method was developed to detect citrus black spot disease (caused by *Phyllosticta citricarpa*) on citrus fruit under field conditions to define site-specific treatments. In both cases, symptoms were pathogen-specific and clearly associated with the disease under study.

A further approach to overcome the elusive nature of the pathogen and to harmonize the phenotyping procedure across experimental sites has been proposed by Russo et al. [39]. The latter developed a phenotyping protocol based on the assessment of the presence of the fungus via real-time PCR in several citrus species. This protocol has been validated by comparing transcriptomic data and the visual assessment of the severity of the symptoms as depicted in Figure 5.

Taking into consideration what reported in the present and the previous paragraphs, tolerance evaluation of the three hybrid populations *ad hoc* constituted is currently performed according to well-defined inoculation and symptoms severity assessment methods, as well as the fungus detection by real-time PCR. To overcome the pitfall of the tracheomycosis phenotyping, the hybrids are currently screened both in vivo and in open-field conditions. The artificial *P. tracheiphilus* inoculation method was developed about 50 years ago [92] and even with the limitations described above, it still remains the most robust, also for early-stage phenotyping [38]. Preliminary field observations of *mal secco* symptoms revealed a clear segregation of tolerance/susceptibility within biological replicates (Figure 5). Finally, *ex planta* or detached leaf methods will be used for a space- and time-effective evaluation of *mal secco* infection.

## 5. Conclusions and Future Perspectives

Much has been done for genetic improvement of lemon to enhance its tolerance to the severe tracheomycosis *mal secco*, but this goal has not yet been achieved due also to the lack of knowledge of the genetic basis of tolerance or resistance. Biotechnological approaches for lemon breeding represented cost- and time-efficient alternatives to traditional breeding, but a complete in-field evaluation of new lemon varieties obtained through in vitro selection, somatic hybridization, and genetic transformation have not yet confirmed their tolerance in the field against *mal secco* disease and have not been diffused for cultivation. Therefore, marker-trait association approaches could represent a useful tool to identify molecular markers associated with tolerance to *mal secco* disease and to perform marker-assisted breeding programs to detect *mal secco* tolerant varieties showing optimal fruit quality.

## Data Availability

All data are contained within the article.
